# Cataract and Amaurosis Produced by Stramonium

**Published:** 1850-09

**Authors:** F. H. Hamilton


ART. II.—*Cataract and Amaurosis produced by Stramonium*, Reported by Prof. F. H. Hamilton.

Mrs. ——— of ———, aged 48, admitted to the Hospital as a private patient, Oct. 23, 1849. When eighteen years old, Mrs. ——— had inflamed eyes, from which she entirely recovered. At the age of 36, a pain commenced in the right eye-ball, which continued at intervals during the four succeeding years. The pain was "heavy," and accompanied with a feeling of fullness and pressure in the ball. This sensation was generally induced by fatigue or excitement, and would subside after a few minutes of quiet. Vision was not in the least degree impaired during this time, except that when the pain existed, the flame of a candle was surrounded by a halo.

During one of these paroxysms of pain, on the 18th of Dec., 1841, she prepared and used as follows :

| | |
|---|---|
| Dry powdered leaves of stramonium, | ℥ j |
| Hot water, | f℥ vij. |

Having steeped this a few minutes, she took a single tea-spoonful of the tea. Immediately after taking it, she was inclined to lie down, and did so. At the end of an hour she got up and felt rather weak, and the lady with whom she was boarding, remarked that her eyes looked very strangely. She now took a second tea-spoonful, which was followed by an increase of weakness, &c. At the third hour, she took the third tea-spoonful, and immediately staggered and fell upon her bed, and felt as if she was dying. She still, however, persisted in taking the medicine; and at the fourth hour, she took the fourth tea-spoonful, immediately after which she became completely blind and paralyzed, and soon lost all consciousness. In this condition she remained four hours, when her consciousness returned, but she had not power to move, or to open her eye-lids. Forty-eight hours after she lifted her lids with her fingers and found she was still totally blind; the light only producing a sensation like the pricking of needles. Those who looked at her eyes now, said there was a "white film over them." Three months from this time, she saw a little light from the side of her eyes, but her eyes continued to pain her, and the light again disappeared. A second time a feeble vision returned, and then finally, about six months after the taking of the Stramonium, her sight became completely and permanently extinguished.

It appears from this record, that Mrs. —— had been threatened with either amaurosis or cataract in one eye for four years previous, showing the existence of a strong predisposition to disease in the organ. It ought, also to be mentioned that on the morning of the day on which she took the stramonium, she had also taken a morphine powder, with valerian and camomile tree; yet, if the account given by herself is correct, (Mrs. —— is a very intelligent and highly respectable lady) of her symptoms immediately preceding each tea-spoonful of the stramonium infusion, no one will doubt but that both the cataract and the amaurosis were produced by the stramonium. It is probable, also, that the cataract was complete at the end of 48 hours, when the friends remarked that she had a "white film over the eyes."

Mrs. —— came to Buffalo for the purpose of having an operation made upon the cataracts. I assured her that such an operation could not possibly give her vision, as there was a complete loss of sensibility in the retina. But, Mrs. —— said, she had her "dark days," and her "light days." On the dark days there was a pitchy darkness before her, and on her light days the darkness seemed to be tempered and less intense. These changes were not dependent upon health, or weather, and occurred indifferently, during the night or the day. She wished me to remove the lenses, and then she could be resigned. I therefore depressed them both in the presence of the Class. The operation was successful, but the retina remained insensible.

